# The WorldWide Antimalarial Resistance Network Clinical Trials Publication Library: A Live, Open-Access Database of *Plasmodium* Treatment Efficacy Trials

**DOI:** 10.4269/ajtmh.19-0706

**Published:** 2020-05-18

**Authors:** Junko Takata, Paul Sondo, Georgina S. Humphreys, Rebekah Burrow, Brittany Maguire, Mohammad S. Hossain, Debashish Das, Robert J. Commons, Ric N. Price, Philippe J. Guerin

**Affiliations:** 1 WorldWide Antimalarial Resistance Network (WWARN), Oxford, United Kingdom;; 2 Infectious Diseases Data Observatory (IDDO), Oxford, United Kingdom;; 3 Institut de Recherche en Sciences de la Santé (IRSS)/Clinical Research Unit of Nanoro (CRUN), Nanoro, Burkina Faso;; 4 Centre for Tropical Medicine and Global Health, Nuffield Department of Clinical Medicine, University of Oxford, Oxford, United Kingdom;; 5 International Centre for Diarrhoeal Disease Research, Bangladesh (icddr,b), Dhaka, Bangladesh;; 6 Global Health Division, Menzies School of Health Research, Charles Darwin University, Darwin, Australia;; 7 Mahidol-Oxford Tropical Medicine Research Unit (MORU), Faculty of Tropical Medicine, Mahidol University, Bangkok, Thailand

## Abstract

Parasite resistance to antimalarial drugs poses a serious threat to malaria control. The WorldWide Antimalarial Resistance Network (WWARN) aims to provide a collaborative platform to support the global malaria research effort. Here, we describe the “WWARN clinical trials publication library,” an open-access, up-to-date resource to streamline the synthesis of antimalarial safety and efficacy data. A series of iteratively refined database searches were conducted to identify prospective clinical trials assessing antimalarial drug efficacy with at least 28 days of follow-up. Of approximately 45,000 articles screened, 1,221 trials published between 1946 and 2018 were identified, representing 2,339 treatment arms and 323,819 patients. In trials from endemic locations, 75.7% (787/1,040) recruited patients with *Plasmodium falciparum*, 17.0% (177/1,040) *Plasmodium vivax*, 6.9% (72/1,040) both, and 0.4% (4/1,040) other *Plasmodium* species; 57.2% (585/1,022) of trials included under-fives and 5.3% (55/1,036) included pregnant women. In Africa, there has been a marked increase in both *P. falciparum* and *P. vivax* studies over the last two decades. The WHO-recommended artemisinin-based combination therapies alone or with a gametocidal drug were assessed in 39.5% (705/1,783) of *P. falciparum* treatment arms and 10.5% (45/429) of *P. vivax* arms, increasing to 78.0% (266/341) and 22.9% (27/118), respectively, in the last five years. The library is a comprehensive, open-access tool that can be used by the malaria community to explore the collective knowledge on antimalarial efficacy (available at https://www.wwarn.org/tools-resources/literature-reviews/wwarn-clinical-trials-publication-library). It is the first of its kind in the field of global infectious diseases, and lessons learnt in its creation can be adapted to other infectious diseases.

## INTRODUCTION

One of the greatest challenges facing malaria control is the ability of *Plasmodium* species to adapt to selective drug pressure. Since World War II, resistance has emerged and spread to all antimalarials that were used en masse in malaria-endemic countries. The development of artemisinin-based combination therapies (ACTs) was an attempt to prevent or slow down antimalarial resistance, but although they remain efficacious first-line treatments in most of the malaria-endemic world, resistance to the artemisinin derivatives and their partner drugs is now emerging.^1^ Synthesizing the available evidence on antimalarial efficacy is essential to understand the drivers of resistance, ensure the optimal use of available treatment options, and facilitate timely and appropriate decision-making by policy-makers.

The WorldWide Antimalarial Resistance Network (WWARN) was established in 2009, with the aim to provide innovative tools and a collaborative data platform to facilitate the optimal use of antimalarial drugs and support the global research effort toward malaria elimination. One such tool is the “WWARN clinical trials publication library,” a comprehensive, systematically constructed database of published antimalarial efficacy trials. The library is actively maintained with periodic updates and can be rapidly searched for relevant studies with pre-extracted data, thus expediting the process of evidence identification and synthesis. To date, this repository has been used to undertake a series of systematic reviews and individual patient data meta-analyses within the WWARN, several of which have resulted in the optimization of antimalarial dosing regimens.^2–9^ Since 2011, the library has been freely available on the WWARN website as an open-access tool for the wider malaria community (https://www.wwarn.org/tools-resources/literature-reviews/wwarn-clinical-trials-publication-library).^10^

Since its inception, the project has evolved, requiring modification of underlying methods and repository format. This article describes the open-source library and the data contained within it, articulates the lessons learnt in its creation, and describes its utility as a resource-efficient model that avoids substantial duplication of effort when tracking antimalarial efficacy trials. The application of this model in other diseases is also explored.

## MATERIALS AND METHODS

### Literature search and study selection.

**Figure 1.**
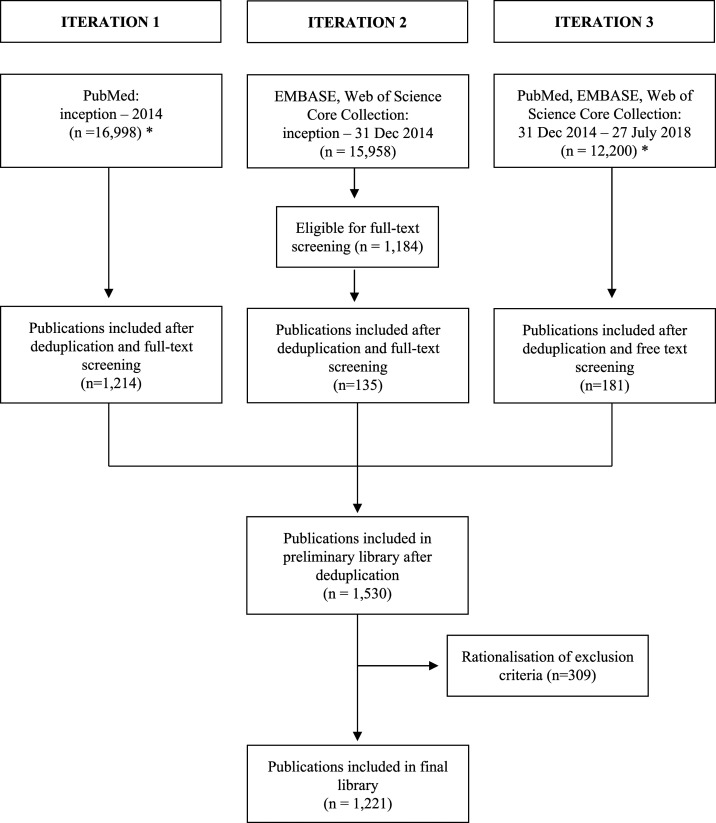
Summary of searches. Iteration numbers correspond to the iterations as described in Supplemental Table 1. For iterations 1 and 3, the numbers denoted by (*) are representative figures computed retrospectively, as summarized in Supplemental Table 5.

Between 2011 and July 2018, a series of searches were conducted in PubMed, Embase, Web of Science Core Collection, and Central databases. Publications were screened by title, abstract, and full text as required. Since 2011, there have been slight variations in the methods used for the searches, screening, and data extraction. These variations include the selection of available databases used in the periodic searches, the time period during which trials were published, the number of reviewers undertaking the research, and the inclusion and exclusion criteria. These variations can be categorized into three main search iterations (documented in Supplemental Tables 1 and 2). The different inclusion and exclusion criteria were then rationalized to the criteria summarized in Table 1. The final library presented in this report is restricted to prospective studies assessing antimalarial drug efficacy to any human-infecting *Plasmodium* species, with a follow-up period of at least 28 days in accordance with the current WHO guidelines.^11^ A summary of the searches is presented in Figure 1.

**Table 1:** Inclusion and exclusion criteria used for the final library

Inclusion criteria	Exclusion criteria
Human-infecting *Plasmodium* species (including mixed infections)	Animal studies
Prospective studies	Prevention or prophylaxis studies
Assessing efficacy of any antimalarial drug	Intermittent preventive treatment studies and intermittent screening and treatment studies in pregnancy
Follow-up of at least 28 days	Mass drug administration
From database inception until the date of most recent search (currently July 27, 2018)	Studies including only severe malaria
Any language	Herbal medicine
	Reviews or pooled analyses
	Case reports
	Conference abstracts
	Asymptomatic/induced malaria studies

### Data extraction.

Variables relating to study design, patient demographics, *Plasmodium* species, antimalarial treatment, and study site were extracted. Initially, data were entered into Microsoft Excel and then subsequently revised using REDCap (Vanderbilt University, Nashville, TN), an electronic data capture tool hosted at the University of Oxford.^12,13^ For non-English language publications, data were extracted fully by a speaker of that language or partially extracted as much as possible by a non-native speaker using translation tools. Data for each treatment arm and study site in a publication were extracted separately and according to species if a publication assessed both *Plasmodium falciparum* and *Plasmodium vivax*. Each study may, therefore, have multiple associated treatments and study sites. Time measures were extracted to the closest year (i.e., year of publication and start and end year of recruitment). The full list and definitions of extracted variables can be found in Supplemental Table 3.

### Data categorization.

Countries were classified into regions and subregions as per the United Nations designation of areas and regions.^14^ Treatments were categorized as follows:

1. Artemisinin-based combination therapies currently recommended by the WHO (artemether–lumefantrine, artesunate–mefloquine, dihydroartemisinin–piperaquine, artesunate–amodiaquine, and artesunate–sulfadoxine–pyrimethamine) or registered to stringent regulatory authorities (artesunate–pyronaridine): all either alone or in combination with gametocidal drugs (primaquine or tafenoquine),
2. any other artemisinin-based therapies,
3. chloroquine,
4. chloroquine in combination with other drugs,
5. quinine,
6. quinine in combination with other drugs, and
7. other.

A full list of all treatment regimens and their classifications can be found in Supplemental Table 4.

### Data analysis.

The primary unit of analysis reported in the Results section is “publication.” The term “study site” is used for each longitude/latitude point on the maps which include all of the individual study sites in one country. The term “study location” is used to infer the country or countries in which a study was conducted rather than the individual study sites. The term “treatment arm” is the unit of analysis for exploring trends in treatment. Reported proportions are calculated using the number of relevant data points available for extraction as the denominator, with the denominators varying according to the number of missing data points. When analyzing time trends, the year 2018 was not included as data were available for only part of the year. Data analysis was conducted in RStudio (version 1.1.463).

## RESULTS

Approximately 45,000 papers were screened to identify a total of 1,221 clinical trials published since 1946 (Figure 1). Overall, 323,819 patients were recruited in 2,339 treatment arms in the study period. Thirty-six studies investigated imported malaria from returning travelers.

### Descriptive characteristics.

Of the 1,185 studies of non-imported malaria, 1,179 (99.5%) recorded location data, of which 524 (44.4%) were conducted in Africa, 523 (44.4%) in Asia, and 97 (8.2%) in South and Central America (Table 2). The species of infection was recorded in 1,040 (87.8%) of studies, with *P. falciparum* assessed in 75.7% (787), *P. vivax* in 17.0% (177), and both species in 6.9% (72) (Table 3). The remaining four (0.4%) studies investigated only other species, that is, *Plasmodium malariae* and *Plasmodium ovale* (together in two studies) and *Plasmodium knowlesi* (two studies). A further 19 studies also assessed *P. malariae*, *P. ovale*, and/or *P. knowlesi* alongside *P. falciparum* and/or *P. vivax*. The majority of studies assessing *P. falciparum* were in Africa (54.1%), whereas those assessing *P. vivax* were mostly undertaken in Asia (67.8%).

**Table 2:** Geographical and language distribution of studies

	Number	%
Region (*n* = 1,179)		
Africa	524	44.4
Northern Africa	28	2.4
Eastern Africa	182	15.4
Western Africa	193	16.4
Middle Africa	84	7.1
Southern Africa	5	0.4
Multi-Africa region	32	2.7
Asia	523	44.4
South Asia	143	12.1
Southeast Asia	324	27.5
East Asia	40	3.4
Western Asia	7	0.6
Multi-Asia region	9	0.8
South and Central America	97	8.2
South America	88	7.5
Central America and Caribbean	8	0.7
Multi-America region	1	0.1
Oceania (Melanesia)	22	1.9
Multi-region	13	1.1
Language (*n* = 1,185)		
English	1,072	90.5
French	57	4.8
Chinese	27	2.3
Portuguese	11	0.9
Spanish	10	0.8
Other	8	0.7

Percentages are derived using the number of studies with available data for that variable as the denominator, denoted by *n*.

**Table 3:** Participant demographics and species assessed, stratified by region

	Asia		Africa		South and Central America		Oceania		Multi-region		Total	
Age of participants (*n* = 1,022)* (years)												
< 5 only	2	1.5%	125	94.0%	0	0.0%	6	4.5%	0	0.0%	133	13.0%
5–15 only	2	14.3%	9	64.3%	0	0.0%	3	21.4%	0	0.0%	14	1.4%
> 15 only	165	67.1%	55	22.4%	22	8.9%	2	0.8%	2	0.8%	246	24.1%
Multiple age categories	299	47.5%	249	39.6%	61	9.7%	9	1.4%	11	1.8%	629	61.5%
Included pregnancy (*n* = 1,036)	18	32.7%	36	65.5%	0	0.0%	1	1.8%	0	0.0%	55	5.3%
Species (*n* = 1,040)												
*P. falciparum*†	303	38.5%	426	54.1%	40	5.1%	6	0.8%	12	1.5%	787	75.7%
*P. vivax*‡	120	67.8%	15	8.5%	37	20.9%	4	2.3%	1	0.6%	177	17.0%
Both§	51	70.8%	3	4.2%	8	11.1%	10	13.9%	0	0.0%	72	6.9%
Other species	3	75.0%	1	25.0%	0	0.0%	0	0.0%	0	0.0%	4	0.4%

*P. falciparum = Plasmodium falciparum*; *P. knowlesi = Plasmodium knowlesi*; *P*. *malariae = Plasmodium malariae*; *P. ovale* = *Plasmodium ovale*; *P*. *vivax = Plasmodium vivax.* Percentages are expressed as a proportion of total studies for each row. Percentages in the final column are derived using the number of studies with available data for that variable as the denominator, denoted by *n*.

* These are mutually exclusive categories, that is, showing studies that were restricted to each age category.

† Includes seven studies that also assessed patients with *P. malariae* and/or *P. ovale*.

‡ Includes one study that also assessed patients with *P. knowlesi*.

§ Includes 11 studies that also assessed patients with *P. malariae*, *P. ovale*, and/or *P. knowlesi*.

One thousand thirty-six (87.4%) studies recorded information on pregnancy status, of which 55 (5.3%) recruited pregnant women; 52 (94.5%) studies were in patients with *P. falciparum* and 36 (65.5%) studies were conducted in Africa. Age information was recorded in 1,022 (86.2%) studies. Children younger than 5 years were included in 585 (57.2%) studies, of which 456 (78.0%) were *P. falciparum* studies and 83 (14.2%) *P. vivax*; 342 (58.5%) studies were conducted in Africa and 189 (32.3%) in Asia. One hundred thirty-three (13.0%) studies were restricted to children younger than 5 years, all but eight of them published after 2000; 124 (93.2%) studies investigated patients with *P. falciparum* which were all undertaken in Africa (Supplemental Table 6a). Although the 124 *P. falciparum* studies restricted to children younger than 5 years constitute only 16.0% (124/774) of all *P. falciparum* trials with available age information, these studies recruited 22.7% (51,039/224,930) of all *P. falciparum* patients and 27.5% (34,766/126,532) of all *P. falciparum* patients treated with a WHO-recommended ACT.

Of 1,039 studies with information on study design, 568 (54.7%) of studies were randomized, of which 95 (16.7%) were at least single blinded. The median duration for study recruitment to the nearest year was 1 year (range: < 1–15 years), and the median time from the end of participant recruitment to publication to the nearest year was 2 years (range: < 1–13 years).

### Temporal trends.

**Figure 2.**
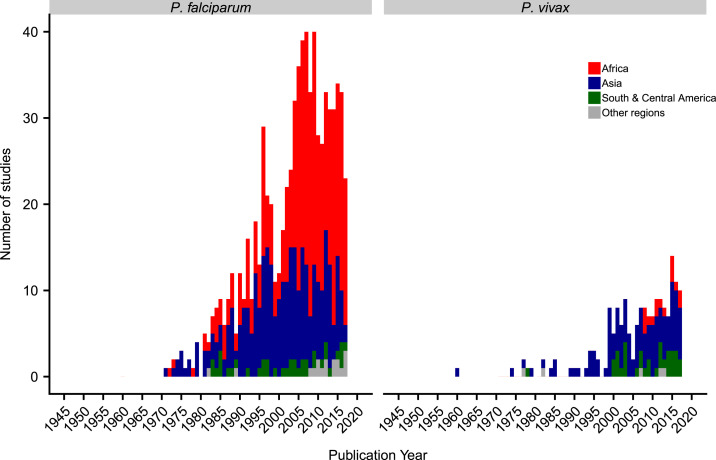
Number of publications per year by region. The year 2018 is not included as data were available for only part of the year. For clarity, the regions of Oceania and multi-region were classified as “other regions.” This figure appears in color at www.ajtmh.org.

The number of antimalarial efficacy trials published per year has increased year on year, until a peak in 2009 for *P. falciparum* and in 2015 for *P. vivax* (Figure 2). Overall, 69.5% (547/787) of *P. falciparum* studies were published on or after 2000, with this increase particularly marked in Africa. There has also been an increase in *P. vivax* studies over time, with 81.9% (145/177) published on or after 2000, including 15 *P. vivax* studies conducted in Africa.

**Figure 3.**
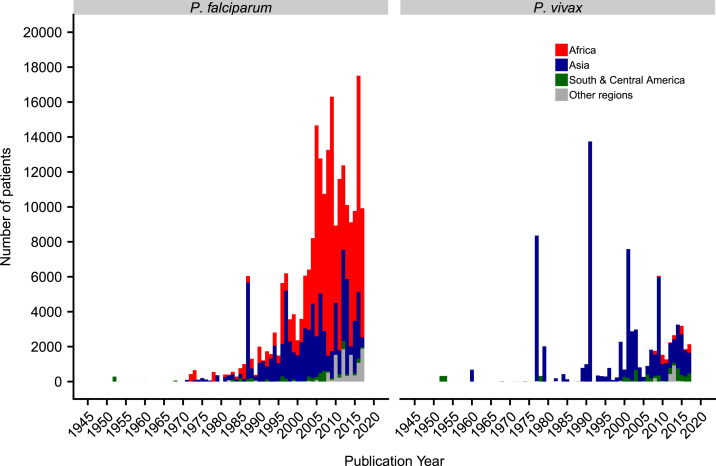
Number of patients recruited per year by region. The year 2018 is not included as data were available for only part of the year. For clarity, the regions of Oceania and multi-region were classified as “other regions.” In *Plasmodium falciparum* studies, the spikes in 1987 and 1997 are due to large studies conducted at the Thai–Myanmar border. In *P. vivax* studies, the spikes in 1977, 1991, 2001, and 2009 were all relapse studies. This figure appears in color at www.ajtmh.org.

The total number of patients recruited per year was similar over time and region in *P. falciparum* studies, although the trend fluctuated for *P. vivax* because of a few very large trials (Figure 3). The median number of patients recruited per treatment arm was 77 (interquartile range (IQR): 40–151) for *P. falciparum* treatment arms and 63 (IQR: 28–125) for *P. vivax.*

### Spatial trends.

Of 1,179 studies with location data, 55 (4.7%) studies recruited patients from multiple countries, 51 (92.7%) of which were conducted after the year 2000. Overall, there were 1,340 study locations across 82 unique countries, of which *P. falciparum* was studied in 931 locations, *P. vivax* in 185, both species in 72, and only other species in 4. For 148 locations, the species were not specified in the text.

**Figure 4.**
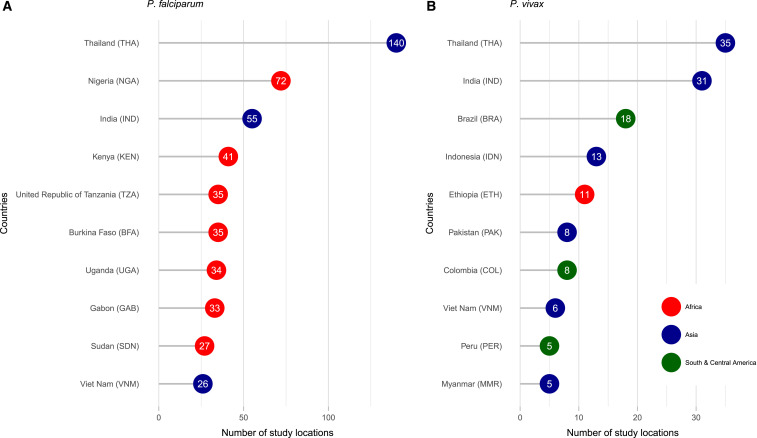
Most common study locations. (**A**) *Plasmodium falciparum*. (**B**) *Plasmodium vivax*. Numbers indicate the number of studies that were located in that country, including multicenter studies. This figure appears in color at www.ajtmh.org.

The greatest number of 931 *P. falciparum* studies was undertaken in Thailand (140, 15.0%), followed by Nigeria (72, 7.7%), India (55, 5.9%), and Kenya (41, 4.4%) (Figure 4). However, in the last 5 years, since 2013, the proportion of studies enrolling patients in Thailand has fallen to 2.6% (6/232) (Supplemental Figure 1), with an associated increase in studies undertaken in Africa. The greatest number of 185 *P. vivax* studies was undertaken in Thailand (35, 18.9%) and India (31, 16.8%); in the last 5 years, there has been a rising proportion of studies from Brazil and Ethiopia.

**Figure 5.**
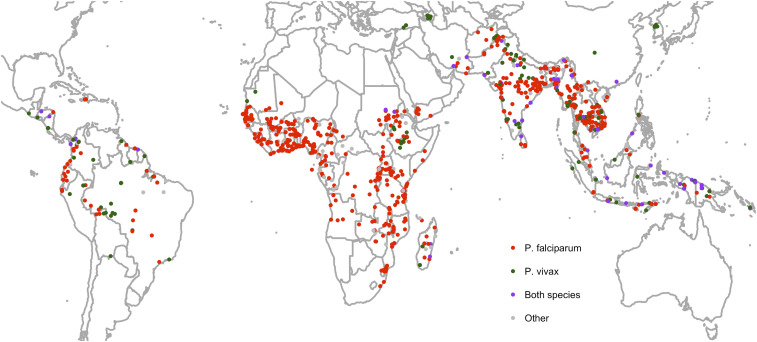
Distribution of study sites. Each dot represents a study site. North America, Europe, and the far north and south have been cropped for clarity as there were no study sites in these areas. This figure appears in color at www.ajtmh.org.

Figure 5 shows the distribution of individual study sites. The most common *P. falciparum* study sites overall were Bangkok, Thailand (*n* = 67), Ibadan, Nigeria (*n* = 44), Mae Sot, Thailand (*n* = 34), and Lambaréné, Gabon (*n* = 29), whereas the most common *P. vivax* study sites were in Bangkok (*n* = 20) and Mae Sot (*n* = 8). The progression in the distribution of study sites is shown in Supplemental Figure 2. Before the 2000s, most studies were focused in Southeast Asia and India. Since the 2000s, there has been a surge in the number of study sites in Africa; in the last 5 years, this has included sites such as Tororo, Uganda (*n* = 8), Kinshasa, Democratic Republic of the Congo (*n* = 7), or Nanoro, Nouna, and Ouagadougou in Burkina Faso (*n* = 6 each). There has also been an increase in *P. vivax* studies in South America and Africa, mainly in Ethiopia.

### Treatments.

**Figure 6.**
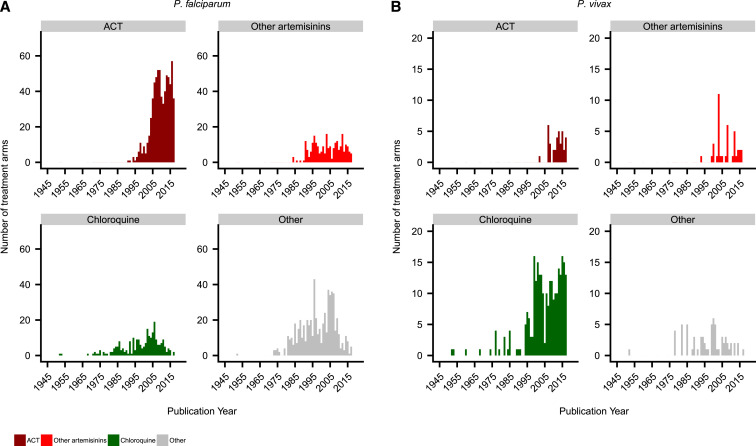
Number of treatment arms per year by drug type. (**A**) *Plasmodium falciparum*. (**B**) *Plasmodium vivax*. The year 2018 is not included as data were available for only part of the year. For clarity, chloroquine in combination with other drugs was included with “chloroquine,” and all quinine combinations were combined with “other.”This figure appears in color at www.ajtmh.org.

**Figure 7.**
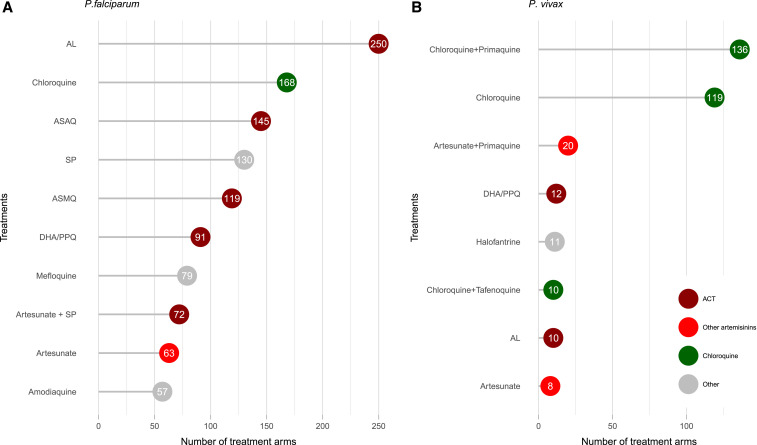
Most common treatment regimens by number of treatment arms. (**A**) *Plasmodium falciparum*. (**B**) *Plasmodium vivax*. Numbers indicate the number of treatment arms that assessed the drug. For clarity, chloroquine in combination with other drugs was included with “chloroquine,” and all quinine combinations were combined with “other.” AL = artemether–lumefantrine; ASAQ = artesunate–amodiaquine; ASMQ = artesunate–mefloquine; DHA/PPQ = dihydroartemisinin–piperaquine; SP = sulfadoxine–pyrimethamine. This figure appears in color at www.ajtmh.org.

**Figure 8.**
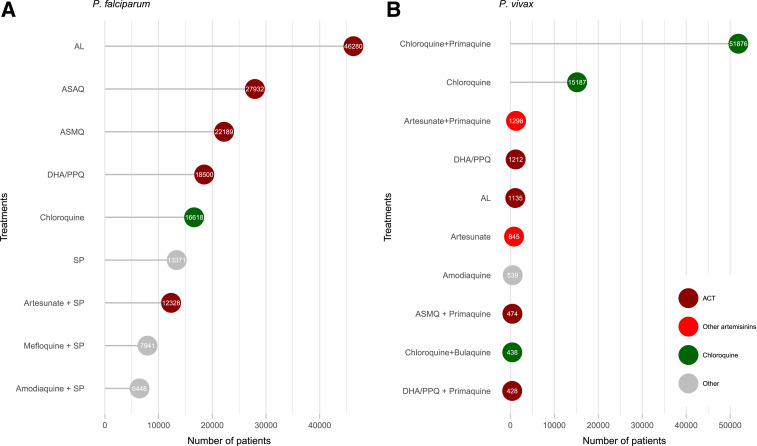
Most common treatment regimens by number of recruited patients. (**A**) *Plasmodium falciparum*. (**B**) *Plasmodium vivax*. Numbers indicate the number of patients who received the drug. For clarity, chloroquine in combination with other drugs was included with “chloroquine,” and all quinine combinations were combined with “other.” AL = artemether–lumefantrine; ASAQ = artesunate–amodiaquine; ASMQ = artesunate–mefloquine; DHA/PPQ = dihydroartemisinin–piperaquine; SP = sulfadoxine–pyrimethamine. This figure appears in color at www.ajtmh.org.

Overall, 2,264 treatment arms were assessed, enrolling 319,167 patients. A total of 236,941 patients with *P. falciparum* were enrolled into 1,783 treatment arms. Artemisinin-based therapies were assessed in 52.9% (944/1,783) of arms, of which 39.5% (705/1,783) included a WHO-recommended ACT, alone or with a gametocidal drug. The number of treatment arms including an ACT has increased substantially over time (Figure 6), particularly in Africa (Supplemental Figure 3). The most commonly studied regimen was artemether–lumefantrine, which was assessed in 14.0% (250/1,783) of treatment arms and 19.5% (46,280/236,941) of patients (Figures 7 and 8). In the last 5 years, since 2013, the number of treatment arms assessing artemisinin-based therapies and WHO-recommended ACTs rose to 88.6% (302/341) and 78.0% (266/341), respectively. There has also been a substantial reduction in the proportion of treatment arms testing non-artemisinin drugs, such as sulfadoxine–pyrimethamine or chloroquine (Supplemental Figure 4).

A total of 78,377 patients with *P. vivax* were enrolled into 429 treatment arms. The predominant antimalarial regimen used was chloroquine, which was assessed either alone or in combination with another drug (excluding artemisinin derivatives) in 64.6% (277/429) of treatment arms. Artemisinin-based therapies were assessed in 85 (19.8%) treatment arms and WHO-recommended ACTs in 45 (10.5%); in the last 5 years, this proportion rose to 29.7% (35/118) and 22.9% (27/118), respectively. Of the 45 treatment arms testing a WHO-recommended ACT, 37.8% (17) were in combination with primaquine.

Nineteen treatment arms assessed 352 patients with mixed *P. falciparum* and *P. vivax* infection (with one arm assessing mixed *P. falciparum*, *P. vivax*, and *P. malariae* infection), of which 10 (52.6%) treatment arms used artemisinin-based therapies and 6 (31.6%) used chloroquine combinations. Sixteen treatment arms assessed 695 patients with other *Plasmodium* species (with one arm assessing mixed *P. malariae* and *P. vivax* infection); chloroquine combinations (62.5%, 10/16) were most commonly used, whereas artemisinin-based therapies were used in 5 (31.2%) treatment arms. The remaining 17 treatment arms assessing 2,802 patients did not specify the species in the text.

### Malaria in returning travelers.

Thirty-six studies investigated malaria in 4,652 returning travelers (Table 4), of which 20 (55.6%) studies were conducted in Western Europe, mostly of travelers returning from sub-Saharan and Western Africa with *P. falciparum*. Twelve studies were undertaken in the United States, all of which enrolled returning military personnel with *P. vivax* most commonly from the Pacific (*n* = 5) or Vietnam (*n* = 3) between 1946 and 1997. Two studies were conducted in Australia and both included military personnel. Of the 75 treatment arms, artemisinin-based therapies were investigated in only four (5.3%).

**Table 4:** Summary of studies in returning travelers

		Species				
Region	Published year(s)	*Plasmodium falciparum*	*Plasmodium vivax*	Both	N/A*	Treatment regimens †
Europe (*n* = 20)‡	1970–2012	13	1	2	4	Quinine combinations (8)
						Mefloquine (8)
						Halofantrine (7)
						Artemether combinations (4)
						Atovaquone combinations (5)
						Chloroquine combinations (2)
United States (*n* = 12)	1946–1997	0	11	1	0	Chloroquine combinations (17)
						Quinine combinations (9)
						Quinacrine combinations (4)
						Primaquine (1)
Japan (*n* = 2)	1986 and 1987	0	0	1	1	Chloroquine (2)
						Quinine (1)
						Sulfadoxine–pyrimethamine (2)
						Sulfamonomethoxine–pyrimethamine (2)
Australia (*n* = 2)	1948 and 2007	1	1	0	0	Quinacrine (1)
						Tafenoquine (1)
						Quinine (1)

* N/A refers to studies in which species data were not specified.

† Five treatment regimens were unknown because of lack of full text.

‡ These consisted of France (9), Germany (3), Denmark (2), Russia (1), United Kingdom (1), and Italy (1). Three were multi-Europe, one of which also included a center in Colombia.

## DISCUSSION

This study highlights the increasing volume of research on antimalarial therapy conducted across the world since the end of World War II. Most studies in the previous century were conducted in Asia, especially in Thailand, but these have declined in recent decades along with the marked reduction of *P. falciparum* malaria in the region. At the same time, there has been a substantial rise in contributions from research centers in sub-Saharan Africa, particularly in clinical trials assessing ACTs for the treatment of *P. falciparum*. According to the 2019 World Malaria Report, 93% of malaria cases occur in the African region, with six countries contributing more than half of all cases worldwide—Nigeria, the Democratic Republic of the Congo, Uganda, Côte d’Ivoire, Mozambique, and Niger.^15^ These countries constituted 17.4% (54/310) of study locations in the last 5 years since 2013 (Supplemental Figure 1), suggesting a reasonable volume of research activity in the most affected countries. Yet, other heavily affected countries such as Rwanda, Guinea, or Central African Republic had very few studies published in the last 5 years compared with, for example, Kenya, which may reflect the distribution of the most active research groups globally. Because of the heterogeneous nature of malaria transmission and resistance, the provision of efficacy data that is spatially and temporally specific is crucial for enabling policy-makers to optimize localized antimalarial policy.

Many trials conducted by national malaria control programs as part of routine surveillance for antimalarial resistance often remain unpublished and unavailable to the scientific community. For those studies which are published, the delay between the end of participant recruitment and publication is often long, with a median of 2 years and a maximum of 13 years in our study, highlighting the continuous challenge of publishing results in a timely manner that can provide policy-makers with contemporaneous data. In the era of increasing resistance of *P. falciparum* to artemisinin and partner drugs, and *P. vivax* to chloroquine, novel ways of reporting and sharing unpublished trial data need to be explored so that the antimalarial resistance landscape can be kept up to date.

There has been a marked rise in the number of studies assessing *P. vivax*, reflecting a growing recognition of the burden of this parasite in Asia, the Americas, and the Horn of Africa. In particular, the recent interest in testing ACTs for *P. vivax* is a reflection of the need to find alternative treatments against chloroquine-resistant parasites and highlights the benefits of developing tools such as the Vivax Surveyor to monitor resistance and identify gaps in our knowledge for this historically less well studied species.^2,16^ Notably, only 23 studies assessed the efficacy of antimalarials in non-falciparum or non-*vivax* species (either alone or alongside *P. falciparum* or *P. vivax*), indicating the limited evidence that currently exists for their treatment.

Children younger than 5 years are particularly vulnerable to malaria, accounting for 67% of malaria deaths worldwide in 2018, and accordingly, 57% of studies in the database included this age-group.^15^ Since the 2000s, there have also been more studies (mainly *P. falciparum* in Africa) that focus specifically on children younger than 5 years. Pregnant women, a similarly vulnerable group, represented just 5% of the total studies published, highlighting a marked lack of evidence in this population.^17^

Finally, whereas some treatments such as ACTs have been studied extensively, some compounds or combinations have been hardly studied. A collation of the current knowledge can, therefore, be used to avoid unnecessary duplication of future effort and provide an easily accessible way to analyze knowledge gaps to guide directions for further research.

Our study has several limitations. The WWARN library is the culmination of iterative refinements since 2011, and so there have inevitably been some discrepancies in methods that would not be the case if the library was created de novo today. It is possible that some studies were missed, especially those published in regional journals or foreign language articles that could not be fully accessed and/or extracted. However, the resources required for such an effort would add comparably little value to the existing resource assembled here. By excluding studies with less than 28 days of follow-up, we have also excluded proportionally more of the earlier published studies than the more recent, which could have contributed to the temporal trends described.

High-quality systematic reviews and meta-analyses are regarded as the highest level in the hierarchy of evidence-based medicine and are used to inform health policy. In practice, conducting reviews that uphold methodological rigor while simultaneously using contemporary knowledge is extremely challenging. A study by Shojania et al. estimated that 7% of systematic reviews fail to incorporate the latest evidence on the day that they are published, and this figure rises to 23% 2 years after publication.^18^ In the rapidly changing field of malaria, the high rates of production of new evidence often exceed the speed with which evidence is synthesized, thus hindering the dissemination of knowledge and translation of knowledge into practice.^19^

In recent years, there have been several innovations that aim to bridge this gap. Systematically constructed, standardized databases can improve efficiency and completeness of systematic reviews, and incorporation of individual patient data allow even greater power to gain new insights.^20,21^ Another emerging concept is that of “living” systematic reviews, which use simplified methods to regularly update reviews and facilitate downstream products such as living guidelines.^22^

The WWARN clinical trials publication library can be used as an upstream contributor to this evolving evidence synthesis landscape, a contemporaneous database of publications, which can be explored easily to understand the extent and nature of knowledge. It will serve as an inventory to inform the availability of data for secondary use, aligning with recent recommendations from policy-makers and scientific journals to support a policy of data sharing.^23–26^ Similar endeavors, termed evidence mapping, have been conducted in other fields, but to our knowledge, this library is the first of its kind in the field of global infectious diseases.^27,28^

Going forward, the library will be updated every 6 months using a search strategy ratified by a systematic review search expert. Results will be screened in specialist software by two independent reviewers, and data extracted by one reviewer and checked by another reviewer. Each update will be recorded in full to enable reporting according to Preferred Reporting Items for Systematic Reviews and Meta-Analyses (PRISMA) guidelines. The standard operating procedure, detailing the search strategy, screening, and extraction methods, will be regularly modified to ensure it captures all relevant publications such as those studying novel antimalarial drugs. With each update, the most recent version of the library with key extracted variables will be made open access on the WWARN website at https://www.wwarn.org/tools-resources/literature-reviews/wwarn-clinical-trials-publication-library. All users will be able to filter and view key variables of relevant studies. The organization of the library will be under continual review, to ensure that it remains easily usable and meets changing needs. It is planned that it will also use novel data visualizations similar to the current WWARN Vivax Surveyor.^16^

Lessons learnt from the WWARN experience are being applied and adapted for other disease themes within the Infectious Diseases Data Observatory, the umbrella platform of WWARN. Search strategies used in previously published systematic reviews on schistosomiasis, soil-transmitted helminths, and visceral leishmaniasis, along with a current review on Chagas disease, will be revised, and an up-to-date search will be planned prospectively every 6 months.^29–31^ Clinical study libraries, similar to the WWARN clinical trials publication library, will be created for each of the aforementioned disease themes and made available as online open-access resources.

## CONCLUSION

In conclusion, this article summarizes the antimalarial efficacy trial landscape since the 1940s and provides a detailed resource for further analyses. The WWARN clinical trials publication library highlights the potential for a novel approach to evidence synthesis and dissemination that can be adapted for other global infectious diseases.

## Supplemental tables and figures

Supplemental materials
