# Supplementary material for: The WorldWide Antimalarial Resistance Network Clinical Trials Publication Library: A Live, Open-Access Database of Plasmodium Treatment Efficacy Trials
Source: Am J Trop Med Hyg. 2020 May 18;103(1):359–68. doi: 10.4269/ajtmh.19-0706 (PMC7356478; doi:10.4269/ajtmh.19-0706)
Supplement: Supplementary file 1 [file tpmd190706.SD1.doc]

**Supplementary Figure 1. Most common study locations in the last five years (2013-2018). A: *P. falciparum*; B: *P. vivax*.**

Numbers indicate the number of studies that were located in that country including multicentre studies. 310 study locations across 60 countries are presented (232 *P. falciparum,* 59 *P. vivax*, 16 both species [not shown] and 3 other species [not shown]).

**Supplementary Figure 2. Distribution of study sites over time.**

Each dot represents a study site. North America, Europe, and the far north and south have been cropped for clarity as there were no study sites in these areas.

**Supplementary Figure 3. Number of treatment arms per year by drug type for Africa and Asia. A: *P. falciparum*, B: *P. vivax*.**

The year 2018 is not included as data were available for only part of the year. For clarity, chloroquine in combination with other drugs were included with “chloroquine”, and all quinine combinations were combined with “other”.

**Supplementary Figure 4. Most common treatment regimens by number of treatment arms in the last five years (2013-2018). A: *P. falciparum*; B: *P. vivax*.**

Numbers indicate the number of treatment arms that assessed the drug. For clarity, chloroquine in combination with other drugs were included with “chloroquine”, and all quinine combinations were combined with “other”. AL = Artemether-Lumefantrine, ASAQ = Artesunate-Amodiaquine, SP = Sulfadoxine-Pyrimethamine, ASMQ = Artesunate-Mefloquine, DHA/PPQ = Dihydroartemisinin-Piperaquine.
