# Supplementary figures and images for: The WorldWide Antimalarial Resistance Network Clinical Trials Publication Library: A Live, Open-Access Database of Plasmodium Treatment Efficacy Trials
Source: Am J Trop Med Hyg. 2020 May 18;103(1):359–68. doi: 10.4269/ajtmh.19-0706 (PMC7356478; doi:10.4269/ajtmh.19-0706)

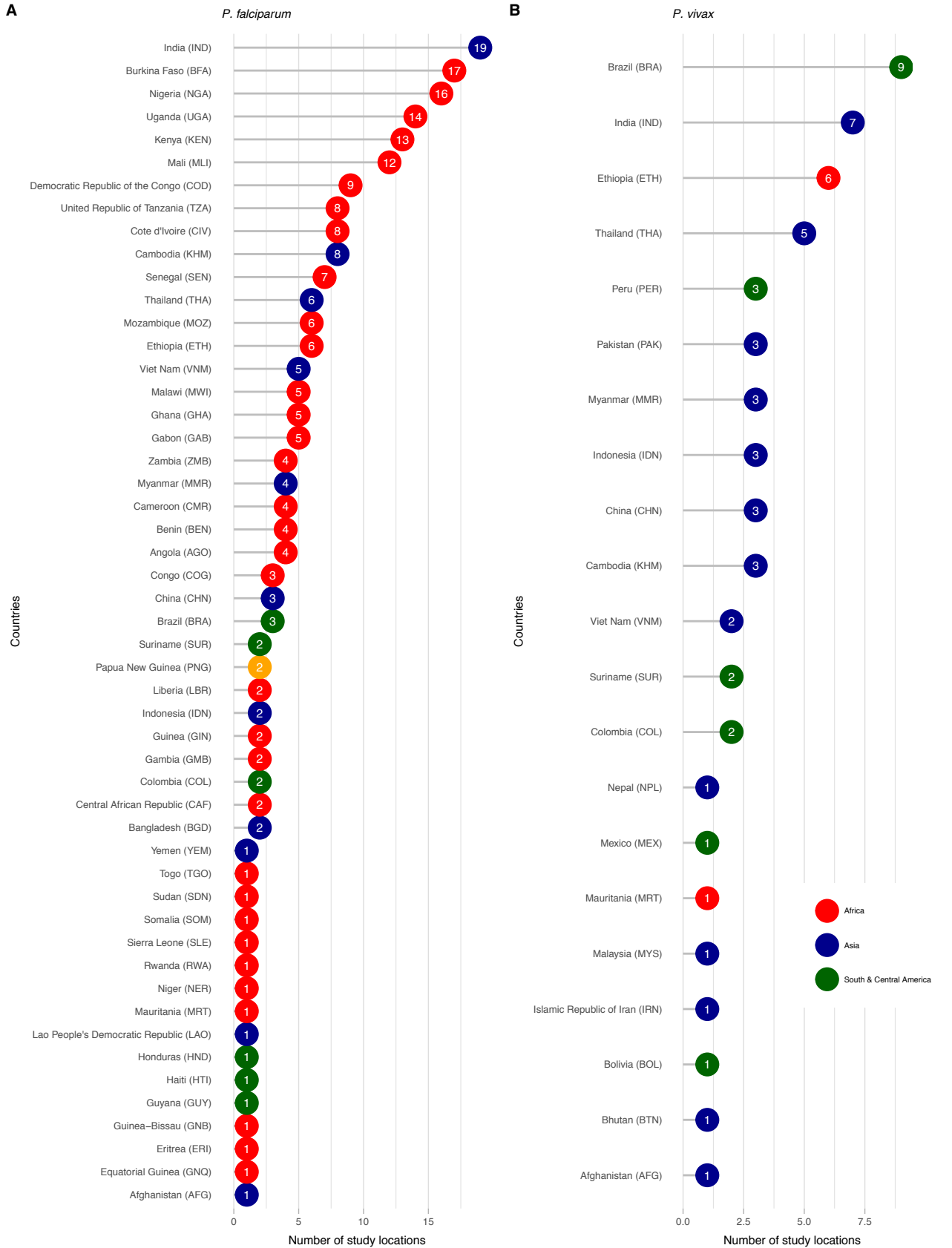

Supplement: Supplementary file 2 [file tpmd190706.SD2.pdf]

Before 1990

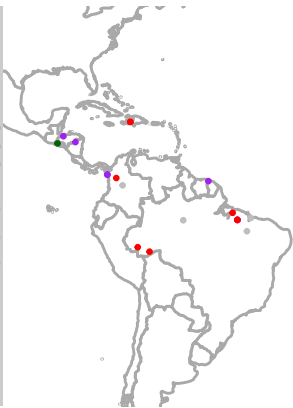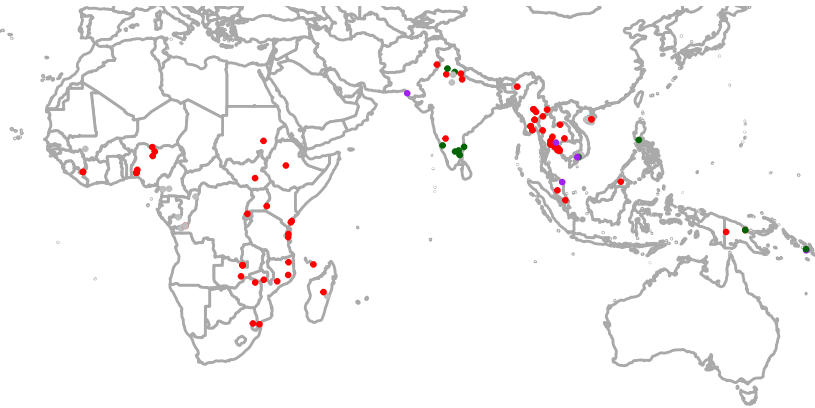

1990s

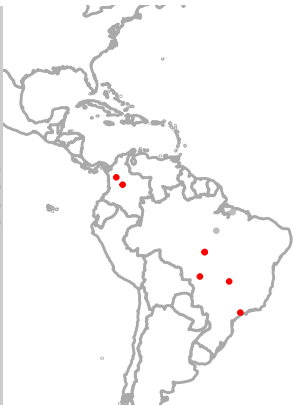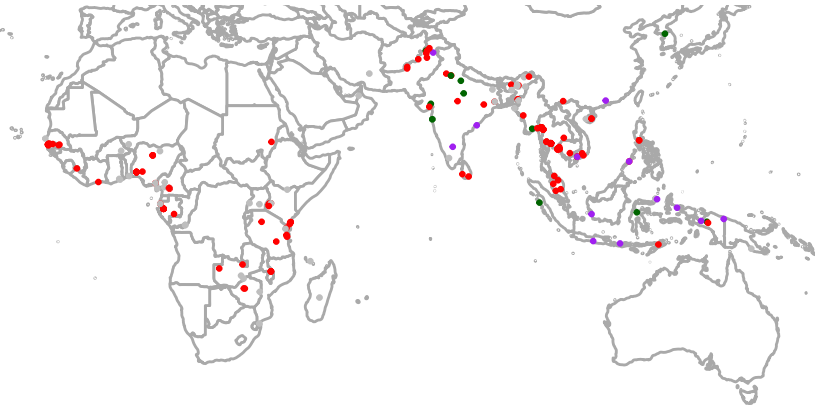

2000s

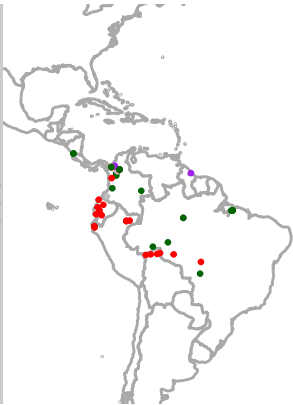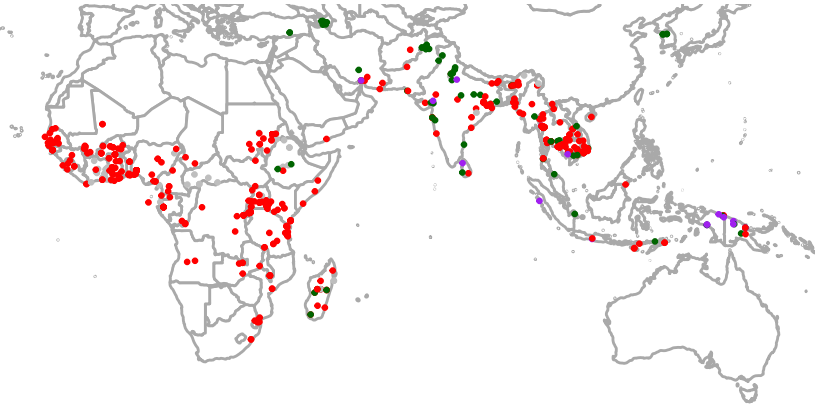

After 2010

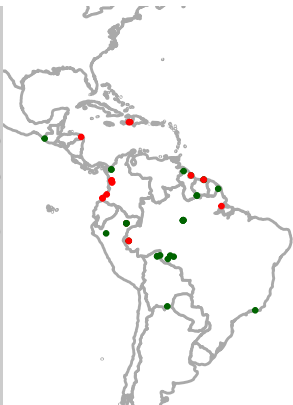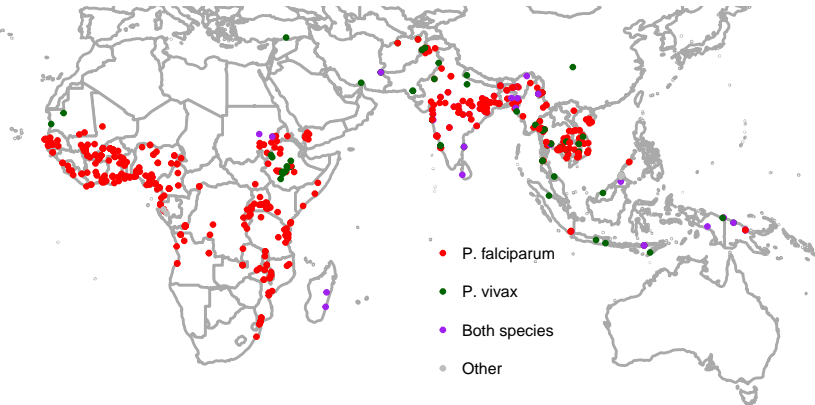

- *P. falciparum*
- *P. vivax*
- Both species
- Other

Supplement: Supplementary file 3 [file tpmd190706.SD3.pdf]

**A***P. falciparum*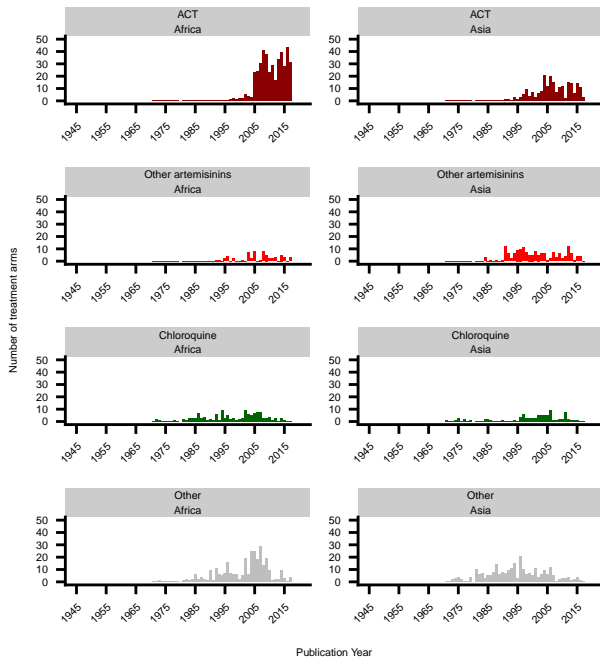**B***P. vivax*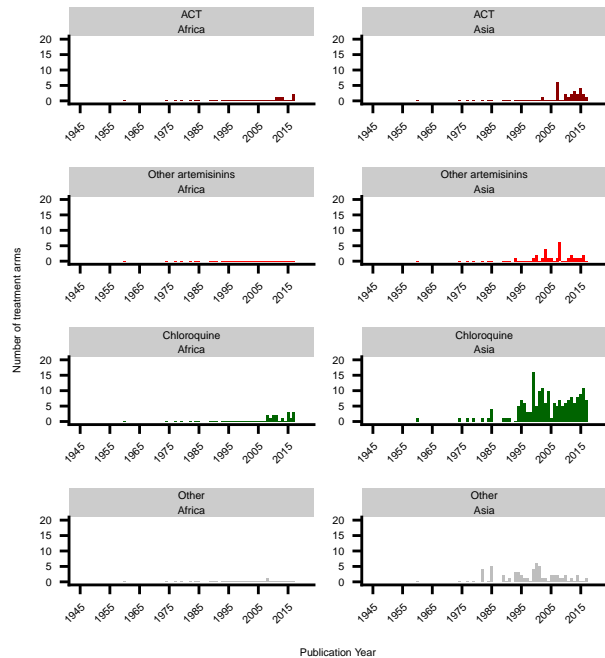

ACT Other artemisinins Chloroquine Other

Supplement: Supplementary file 4 [file tpmd190706.SD4.pdf]

**A***P. falciparum*

Treatments

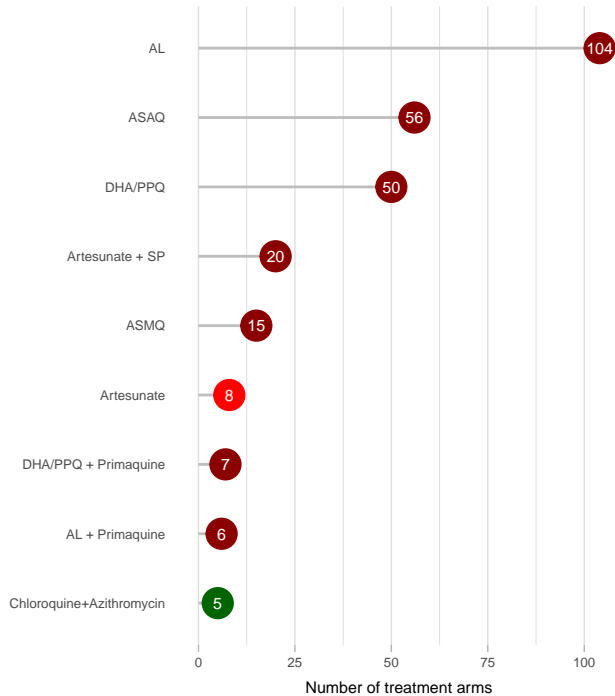**B***P. vivax*

Treatments

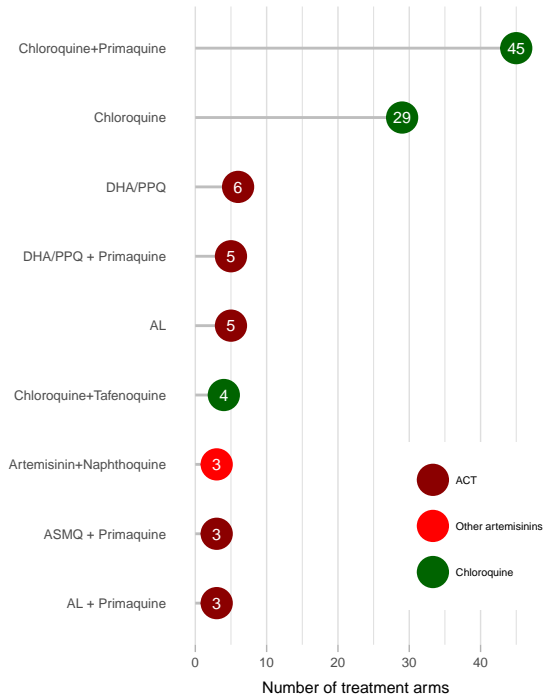

Supplement: Supplementary file 5 [file tpmd190706.SD5.pdf]
