# Supplementary material for: The WorldWide Antimalarial Resistance Network Clinical Trials Publication Library: A Live, Open-Access Database of Plasmodium Treatment Efficacy Trials
Source: Am J Trop Med Hyg. 2020 May 18;103(1):359–68. doi: 10.4269/ajtmh.19-0706 (PMC7356478; doi:10.4269/ajtmh.19-0706)
Supplement: Supplementary file 6 [file tpmd190706.SD6.doc]

**Supplementary Table 1: Summary of search iterations.**

|  | Iteration 1 | Iteration 2 | Iteration 3 |
| --- | --- | --- | --- |
| Database searched | Pubmed | EMBASE, Web of Science Core Collection | Pubmed, EMBASE via Ovid, Web of Science Core Collection, CENTRAL ** |
| Screening method | Single screened | Single screened | 2015-17: eligible studies checked by second reviewer  2017-18: double screened by two reviewers |
| Search period | Inception – 31 Dec 2014 | Inception – 31 Dec 2014 | 1 Jan 2015 – 27^th^ July 2018 |
| Search terms * | A | A *OR* B | A *OR* B *** |
| Inclusion criteria | Prospective efficacy studies  Any human-infecting *Plasmodium*  Any length of follow-up | Prospective efficacy studies  Any human-infecting *Plasmodium*  Any length of follow-up | Prospective efficacy studies  Any human-infecting *Plasmodium*  ≥28 days follow-up |
| Exclusion criteria | Prevention/prophylaxis studies  Intermittent screening and treatment  Intermittent preventative treatment  Reviews and pooled analyses  Severe malaria only  Animal studies  Herbal medicine | *As with Iteration 1, plus*  Mass drug administration  Asymptomatic / volunteer studies  Conference abstracts  Case reports | *As with Iteration 1, plus*  Mass drug administration  *But not*  Herbal medicine |

The series of searches can be summarized into the 3 iterations above.

* Search terms are summarized in Supplementary Table 2.

** CENTRAL database was only searched between 1 Jan 2017 – 5 Jan 2018.

*** Between 1 Jan 2015 – 31 Dec 2016, both A and B search terms were modified to include the drugs artefenomel and arterolane.

**Supplementary Table 2: Search terms.**

| A) | (malaria *OR* plasmodium) | *AND* | (amodiaquine OR atovaquone OR artemisinin OR arteether OR artesunate OR artemether OR artemether OR artemotil OR azithromycin OR artekin OR chloroquine OR chlorproguanil OR cycloguanil OR clindamycin OR coartem OR dapsone OR dihydroartemisinin OR duo-cotecxin OR doxycycline OR halofantrine OR lumefantrine OR lariam OR malarone OR mefloquine OR naphthoquine OR naphthoquinone OR piperaquine OR primaquine OR proguanil OR pyrimethamine OR pyronaridine OR proguanil OR quinidine OR quinine OR riamet OR sulphadoxine OR tetracycline OR tafenoquine)) | | | | |
| --- | --- | --- | --- | --- | --- | --- | --- |
| B) | (malaria *OR* plasmodium) | *AND* | (resistance OR susceptibility OR sensitivity) | *AND* | (in vitro OR ex vivo) | *AND* | (amodiaquine OR atovaquone OR artemisinin OR arteether OR artesunate OR artemether OR artemether OR artemotil OR azithromycin OR artekin OR benflumetol OR chloroquine OR chlorproguanil OR cycloguanil OR clindamycin OR coartem OR dapsone OR desethylamodiaquine OR dihydroartemisinin OR duo-cotecxin OR doxycycline OR halofantrine OR lumefantrine OR lariam OR malarone OR mefloquine OR monodesethylamodiaquine OR naphthoquine OR naphthoquinone OR piperaquine OR proguanil OR pyrimethamine OR pyronaridine OR proguanil OR quinine OR riamet OR sulphadoxine OR tetracycline) |

See Supplementary Table 1 for details on how these were combined or modified with each iteration. All search terms were searched in All Fields in Pubmed, .mp (keywords) in EMBASE via OVID, and TS (topic) in Web of Science Core Collection.

**Supplementary Table 3. List of extracted variables**

| Worksheet | Variable | Format | Definitions and comments |
| --- | --- | --- | --- |
| Register data | Authors | Free text | Auto-populated |
|  | Year of publication | Free text | Auto-populated |
|  | Title | Free text | Auto-populated |
|  | Journal name, volume, issue number, page | Free text | Auto-populated |
|  | Region of study | Africa, Asia, Central America, Europe, North America, South America, Multiregional, Oceania |  |
|  | Country of study | All countries, or multi-center |  |
|  | Language of full text | English, French, Chinese, Spanish, Portuguese, Other (free text) |  |
| Study data | First and last year of recruitment | Free text |  |
|  | Randomization | Yes/No/Not specified |  |
|  | Blinded | Yes/No/Not specified | Recorded as ‘yes’ if at least single-blinded. |
|  | Participant information   - Under 5 years - 5-15 years - 15 years or older - Pregnant women | Yes/No/Not specified, per category | Recorded as ‘yes’ if those participants were included; more than one category could be selected ‘Yes’. |
|  | Length of follow-up in days | Free text | If multiple lengths of follow-up (e.g. per drug), the longest duration was recorded. |
|  | Species information   - *P. falciparum* - *P. vivax* - *P. ovale* - *P. malariae* - *P. knowlesi* - Mixed species | Yes/No/Not specified per category | Recorded as ‘yes’ if the species were included; more than one category could be selected ‘Yes’. |
| Treatment data | Species assessed in treatment arm | Pf, Pv, All, Other (free text) | If there were multiple species tested in a study, each arm per species was recorded as a separate entry and the species specified here as either: Pf (*P. falciparum*), Pv (*P. vivax*), All (unspecified whether Pf or Pv), or Other (mixed species, other *Plasmodium* species, etc). |
|  | Name of drug in treatment arm | Free text | If multiple treatment arms, each treatment arm was recorded as a separate entry. |
|  | Number of participants per arm | Free text | The number of patients enrolled per arm, prior to any losses to follow-up. |
| Site data | Species assessed at study site | Pf, Pv, All, Other (free text) | If there were multiple species tested in a study, each study site per species was recorded as a separate entry and the species specified here as either: Pf (*P. falciparum*), Pv (*P. vivax*), All (unspecified whether Pf or Pv), or Other (mixed species, other *Plasmodium* species, etc). |
|  | Country of study site | All countries | If there were multiple study sites per study, each site was recorded as a separate entry. |
|  | Location of study site | Free text | If a specific location was not identified, the capital city was recorded. |
|  | Longitude and latitude of study site | Free text | Longitude and latitude of the location recorded above. Google Maps was used if not reported in the publication. |

**Supplementary Table 4: Classification of treatment regimens.**

| **Classification** | **Treatment regimens** | **Number of arms** |
| --- | --- | --- |
|  |  | ***Total*** |
| **ACTs** | Artemether-Lumefantrine | 266 |
| *(currently recommended by WHO, or registered to stringent regulatory authorities)* | Artesunate + Sulfadoxine-Pyrimethamine | 77 |
|  | Artesunate-Amodiaquine | 150 |
|  | Artesunate-Mefloquine | 120 |
|  | Artesunate-Pyronaridine | 7 |
|  | Dihydroartemisinin-Piperaquine | 108 |
|  | ***TOTAL*** | ***728*** |
| **ACTs + gametocidal drug** | Artemether-Lumefantrine + Primaquine | ***11*** |
|  | Artesunate + Sulfadoxine-Pyrimethamine + Primaquine | ***4*** |
|  | Artesunate-Amodiaquine + Primaquine | ***2*** |
|  | Artesunate-Mefloquine + Primaquine | ***8*** |
|  | Artesunate-Pyronaridine + Primaquine | ***1*** |
|  | Dihydroartemisinin-Piperaquine + Primaquine | ***13*** |
|  | ***TOTAL*** | ***39*** |
| **Other artemisinin-based therapies** | Arteether | 3 |
|  | + Sulfadoxine-Pyrimethamine | 1 |
|  | Artefenomel |  |
|  | + Piperaquine | 1 |
|  | + Chloroquine + Primaquine | 1 |
|  | + Artesunate + Mefloquine | 1 |
|  | Arteflene | 1 |
|  | Artemether | 23 |
|  | + Azithromycin | 1 |
|  | + Doxycycline | 1 |
|  | + Mefloquine | 15 |
|  | + Pyrimethamine | 3 |
|  | Artemether-Lumefantrine |  |
|  | + Artesunate | 2 |
|  | + Quinine | 1 |
|  | Artemisinin | 13 |
|  | + Doxycycline | 1 |
|  | + Mefloquine | 3 |
|  | + Mefloquine + Sulfadoxine-Pyrimethamine | 1 |
|  | + Naphthoquine | 15 |
|  | + Piperaquine | 9 |
|  | + Quinine | 1 |
|  | + Sulfadoxine-Pyrimethamine | 1 |
|  | + Tetracycline | 1 |
|  | Arterolane | 6 |
|  | + Piperaquine | 4 |
|  | + Piperaquine + Primaquine | 1 |
|  | Artesunate | 72 |
|  | + Amodiaquine + Chlorpheniramine | 1 |
|  | + Amodiaquine + Methylene Blue | 2 |
|  | + Artesunate-Mefloquine | 1 |
|  | + Atovaquone-Proguanil | 5 |
|  | + Azithromycin | 6 |
|  | + Chloroquine | 10 |
|  | + Chlorproguanil-Dapsone | 6 |
|  | + Clindamycin | 2 |
|  | + Co-trimoxazole | 1 |
|  | + Dihydroartemsinin-Piperaquine | 2 |
|  | + Doxycycline | 3 |
|  | + Ferroquine | 4 |
|  | + Fosmidomycin | 5 |
|  | + Lumefantrine | 2 |
|  | + Mefloquine + KAF156 | 1 |
|  | + Methylene Blue | 1 |
|  | + Primaquine | 22 |
|  | + Pyrimethamine | 1 |
|  | + Quinine | 2 |
|  | + Sulfamethoxypyrazine-Pyrimethamine | 6 |
|  | + Tetracycline | 1 |
|  | Dihydroartemisinin | 4 |
|  | + Azithromycin | 1 |
|  | + Mefloquine | 7 |
|  | + Napthoquine | 1 |
|  | + Napthoquine -Trimethoprim | 1 |
|  | + Piperaquine-Trimethoprim | 4 |
|  | + Piperaquine-Trimethoprim + Primaquine | 1 |
|  | ***TOTAL*** | ***285*** |
| **Chloroquine** | Chloroquine | ***305*** |
| **Chloroquine + other** | Chloroquine |  |
|  | + Atovaquone-Proguanil | 1 |
|  | + Azithromycin | 7 |
|  | + Bulaquine | 2 |
|  | + Chlorpheniramine | 4 |
|  | + Chlorpheniramine + Sulfadoxine-Pyrimethamine | 1 |
|  | + Clindamycin | 3 |
|  | + Doxycycline | 5 |
|  | + Elubaquine | 1 |
|  | + Erythromycin | 1 |
|  | + Ketotifen | 1 |
|  | + Primaquine | 143 |
|  | + Pyrimethamine | 4 |
|  | + Simvastatin | 2 |
|  | + Sulfadoxine-Pyrimethamine + Primaquine | 2 |
|  | + Sulfadoxine-Pyrimethamine | 41 |
|  | + Tafenoquine | 10 |
|  | + Tetracyline | 1 |
|  | + Primaquine + KAF156 | ***1*** |
|  | + Verapamil | ***1*** |
|  | ***TOTAL*** | ***231*** |
| **Quinine** | Quinine | ***57*** |
| **Quinine + other** | Quinine |  |
|  | + Allopurinol | 1 |
|  | + Azithromycin | 5 |
|  | + Chloroquine | 2 |
|  | + Clindamycin | 9 |
|  | + Co-trimoxazole | 1 |
|  | + Doxycycline | 6 |
|  | + Mefloquine | 1 |
|  | + Primaquine | 3 |
|  | + Pyrimethamine | 1 |
|  | + Quinidine-Cinchonin | 5 |
|  | + Robeprazole | 1 |
|  | + Spiramycin | 1 |
|  | + Sulfadoxine | 1 |
|  | + Sulfadoxine-Pyrimethamine | 10 |
|  | + Tetracycline | 18 |
|  | ***TOTAL*** | ***65*** |
| **Other** | Amodiaquine | 64 |
|  | + Co-trimoxazole | 1 |
|  | + Methylene Blue | 1 |
|  | + Primaquine | 2 |
|  | + Simvastatin | 1 |
|  | + Sulfadoxine | 1 |
|  | + Sulfadoxine-Pyrimethamine | 38 |
|  | + Sulfalene-Pyrimethamine | 4 |
|  | + Tetracycline | 1 |
|  | AQ-13 | 1 |
|  | Atovaquone | 2 |
|  | + Doxycycline | 1 |
|  | + Pyrimethamine | 2 |
|  | + Tetracycline | 1 |
|  | Atovaquone-Proguanil | 22 |
|  | + Primaquine | 1 |
|  | Azithromycin | 3 |
|  | + Primaquine | 1 |
|  | Chlorproguanil | 1 |
|  | + Dapsone | 10 |
|  | Clindamycin | 7 |
|  | Cotrimoxazole | 7 |
|  | Doxycycline | 3 |
|  | Ferroquine | 1 |
|  | Fosmidomycin | 1 |
|  | + Clindamycin | 5 |
|  | + Piperaquine | 1 |
|  | Halofantrine | 45 |
|  | + Primaquine | 3 |
|  | KAE609 (cipargamin) | 2 |
|  | KAF156 | 1 |
|  | Mefloquine | 87 |
|  | + Doxycyline | 1 |
|  | + Primaquine | 4 |
|  | + Sulfadoxine-Pyrimethamine | 33 |
|  | + Sulfadoxine-Pyrimethamine + Primaquine | 1 |
|  | + Sulfadoxine-Pyrimethamine + Tetracycline | 1 |
|  | + Tetracycline | 2 |
|  | Metakelfin | 1 |
|  | Methylene blue | 1 |
|  | Norfloxacin | 2 |
|  | Pafuramidine | 1 |
|  | + Primaquine | 1 |
|  | Primaquine | 7 |
|  | Proguanil | 5 |
|  | + Dapsone | 1 |
|  | Pyrimethamine | 1 |
|  | + Dapsone | 1 |
|  | + Diformyldapsone | 1 |
|  | Pyronaridine | 2 |
|  | Quinidine | 1 |
|  | Rifampicin | 1 |
|  | + Primaquine | 1 |
|  | SAR97276A | 1 |
|  | Sulfadiazine-Pyrimethamine | 1 |
|  | Sulfadoxine-Pyrimethamine | 138 |
|  | + Azithromycin | 1 |
|  | + Erythromycin | 1 |
|  | + Piperaquine | 1 |
|  | + Primaquine | 3 |
|  | + Probenecid | 1 |
|  | + Pyronaridine | 1 |
|  | + Simvastatin | 1 |
|  | Sulfalene-Pyrimethamine | 4 |
|  | Sulfamethoxazole + Proguaxnil | 1 |
|  | Tafenoquine | 1 |
|  | Tetracycline | 1 |
|  | Wr 33063 | 1 |
|  | ***TOTAL*** | ***547*** |

**Supplementary Table 5: Retrospective search conducted to create Figure 1.**

| Iteration | Database searched | Dates | Search terms | Number of hits | Total hits per iteration |
| --- | --- | --- | --- | --- | --- |
| 1 | Pubmed | Inception – 31/12/14 | A | 16998 | 16998 |
| 2* | N/A | N/A | N/A | N/A | N/A |
| 3 | Pubmed | 01/01/15 - 31/12/16  01/01/17 – 27/07/18 | A *OR* B **  A *OR* B | 1849  1663 | 12200 |
|  | Web of Science Core Collection | 01/01/15 - 31/12/16  01/01/17 – 27/07/18 | A *OR* B **  A *OR* B | 2293  1809 |  |
|  | EMBASE via Ovid | 01/01/15 - 31/12/16  01/01/17 – 27/07/18 | A *OR* B **  A *OR* B | 3227  1273 |  |
|  | CENTRAL | 01/01/17 – 05/01/18 | A *OR* B | 86 |  |

Searches were conducted on 06/03/2019 in the respective databases above, using the dates and search terms per iteration as described in Supplementary Tables 1 and 2. The values in the final column are used in Figure 1 in the main text, as an illustrative figure to convey the magnitude of work involved in the searches, as the original numbers were not recorded contemporaneously. All search terms were searched in All Fields in Pubmed, .mp (keywords) in EMBASE via OVID, and TS (topic) in Web of Science Core Collection.

* No retrospective search was required for iteration 2, as the figures at each step were recorded contemporaneously.

** As noted in Supplementary Table 1, the search terms for these dates were modified to include the drugs artefenomel and arterolane.
